# A novel lupene derivative from *Thymus capitatus* possesses an apoptosis-inducing effect via Let-7 miRNA/Cyclin D1/VEGF cascade in the A549 cell line

**DOI:** 10.1186/s12906-023-04201-7

**Published:** 2023-10-16

**Authors:** Nora M. Aborehab, Maha M. Salama, Shahira M. Ezzat

**Affiliations:** 1 Department of Biochemistry, Faculty of Pharmacy, October University for Modern Sciences and Arts (MSA), Giza, 12451 Egypt; 2 Department of Pharmacognosy, Faculty of Pharmacy, Cairo University, Kasr El-Ainy St., Cairo11562, Cairo, 11562 Egypt; 3 Department of Pharmacognosy, Faculty of Pharmacy, The British University in Egypt, Suez Desert Road, El Sherouk City, Cairo, 11837 Egypt; 4 Department of Pharmacognosy, Faculty of Pharmacy, October University for Modern Sciences and Arts (MSA), Giza, 12451 Egypt

**Keywords:** *Thymus capitatus* L., Lupeol derivatives, A549 human non-small cell lung cancer cell line, miRNA-21, Let-7 miRNA

## Abstract

Non-small-cell lung carcinoma (NSCLC) is a type of epithelial lung cancer accounting for about 85% of all lung cancers. In our research, a novel lupene derivative namely acetoxy-lup-5(6), 20(29)-diene (ALUP), as well as two known triterpenes; lupeol (LUP) and betulinic acid (BA) were isolated through the chromatographic purification of the 95% ethanolic extract of *Thymus capitatus*. Identification of the compounds was carried out by physicochemical properties as well as spectral 1D and 2D NMR analysis. The anti-cancer activity of the three triterpenes was assessed on non-small cell lung cancer cell line; A549 using MTT assay and cell cycle analysis using annexin V/propidium iodide. The molecular mechanism underlying anti-apoptotic effects was determined by analyzing Let-7 miRNA and miRNA-21 expression, the mRNA gene expression level of Bax, CASP-8, CD95, Bcl2, KRAS, VEGF, Cyclin D1 using qRT-PCR. Our results revealed that the three isolated compounds ALUP, LUP, and BA caused cell cycle arrest at the G2/M phase with an increase in the apoptosis which may be attributed to their significant effect on raising Bax, CASP-8, and CD95 and reducing the mRNA expression levels of Bcl-2, KRAS, VEGF, and Cyclin D1 compared to control cells. RT-PCR results showed that the ALUP, LUP, and BA significantly downregulated miRNA-21 expression. Meanwhile, the three compounds caused significant overexpression of Let-7 miRNA. This is the first report on the anti-cancer activity of acetoxy-lup-5(6), 20(29)-diene (ALUP) in reducing the proliferation and differentiation of the A549 cell line through inducing apoptosis. Finally, by targeting the Let-7 miRNA/Cyclin D1/VEGF cascade, acetoxy-lup-5(6), 20(29)-diene could be a potential therapeutic agent for lung cancer.

**Supplementary Information:**

The online version contains supplementary material available at 10.1186/s12906-023-04201-7.

## Introduction

Lung cancer is ranked as the second principal cause of cancer death worldwide [1]. About 25% or more of cancer deaths are due to lung cancer. Cancer cells are divided into small-cell lung cancer (SCLC) and non-small-cell lung cancer (NSCLC).

NSCLC accounts for about 85% of lung cancer diagnoses, however, it progresses at a slower rate than SCLC accompanied by minor signs until it aggravates. The sub-categories of NSCLC are adenocarcinoma of the lung, squamous cell, and large-cell undifferentiated carcinoma [2]. Adenocarcinoma is a type of lung cancer, accounting for 30% of all cases and responsible for 40% of all NSCLC prevalence [3].

Due to the severe side effects of chemotherapy and radiotherapy in cancer treatment, where non-selectivity is one of their major drawbacks, additionally, multi-drug resistance is a massive challenge in treatment. The search for novel strategies in cancer management is the researchers’ priority nowadays. Since more than 60% of anticancer compounds are isolated from herbal, marine, and microorganism sources, therefore, accessibility of natural products with high efficacy and low adverse effects is vital [4]. Moreover, by adjusting the multimolecular targets of natural products that are involved in angiogenesis, metastasis, and multidrug resistance, natural products could be applied as potentially effective drugs in the prevention of lung cancer.

Among the promising natural products that have anticancer effects are triterpenoids which have been reported to exhibit anti-proliferative activity when tested against different types of cancer cell lines [5]. Moreover, many reports showed that triterpenes have in vitro and in vivo inhibitory effects on tumor growth, and cell cycle progression, and they could also induce apoptosis of cancer cells. These triterpenoids belong to different classes such as, lupane type [6].

*Thymus capitatus* (L.) Hoffmanns. & Link (the plant of our interest), conehead thyme, Persian-hyssop, and Spanish oregano, belong to the family Lamiaceae. It is native to the Mediterranean region and distributed in the western Mediterranean region of Egypt [7]. Most of the studies focused on the essential oil composition of *Thymus capitatus* and its antibacterial and antifungal properties as well as antioxidant properties [8–10]. In addition to, their reported antinociceptive activity [11], cytotoxic, and colon pathogen adhesion-inhibition properties [12]. Phytochemical characterization of *Thymus capitatus* recorded that the plant is rich in triterpenoids, flavonoids, and phenolic acids [13]. Few reports dealt with the phytochemical extraction and isolation of the bioactive triterpenes from *T. capitatus* and their anticancer effect.

Micro-RNA-21 (miRNA-21) is a specific miRNA that is overexpressed in almost all solid tumors such as prostate, pancreas, breast, and lung cancers. Bcl2 (B-cell lymophoma 2)has an efficient role in the apoptosis pathway, it regulates the activity of caspases through cytochrome C sequestering in the mitochondria *via* inhibiting of protein Bax (Bcl2-Associated X Protein) [14]. On the other hand, another study indicates that miRNA-21 may downregulate Bax and upregulate Bcl2, thus inhibiting apoptosis [15].

Let-7 miRNA was reported to directly suppress Cyclin D1-associated signals, with subsequent downregulation of Bcl2 and upregulation of Bax as well as caspase-3, caspase-8, and caspase-9. This cascade negatively affects cell growth and triggers cell apoptosis [16].

Angiogenesis is a characteristic of tumor growth, where the newly created blood channels enable waste products to be simultaneously removed along with the diffusion of nutrients and oxygen to exhausted peripheral tumor cells, promoting tumor cell survival and tumor growth. The angiogenic process is regulated at various levels, starting with factors within the tumor microenvironment, followed by the modifications in signaling pathways containing key regulatory molecules as vascular endothelial growth factor (VEGF) expression [17].

Therefore, this study is designed to isolate the major triterpenes from the ethanolic extract of *T. capitatus* in order to investigate their underlying mechanism in inducing apoptosis via the regulation of Let-7 miRNA/Cyclin D1/VEGF cascade in non-small cell lung carcinoma (NSCLC).

## Experimental procedures

### Extraction and isolation

#### Plant material

The aerial parts of *Thymus capitatus* (L.) Hoffmanns. & Link were collected from North Coast, Egypt in the Spring of 2019. The plant was authenticated in the Herbarium of the Faculty of Science, Cairo University. A voucher specimen (6-05-2019) was kept in the herbarium of the Pharmacognosy Department, Faculty of Pharmacy, Cairo University, Cairo, Egypt, with a deposition number (TC-00107).

#### Extraction

The dried powdered aerial parts of *T. capitatus* (500 gm) were extracted with 95% ethanol by cold percolation (3 L three times, each for 3 days) till exhaustion. The combined filtered ethanolic extract was evaporated under reduced pressure using a rotary evaporator at 40ºC to yield 75 gm of dried residue. The plant experiments were performed in accordance with relevant guidelines and regulations.

### Materials for isolation

Silica gel 60 (70–230 mesh ASTM; Fluka, Steinheim, Germany). Thin-layer chromatography (TLC) was performed on silica gel GF_254_ precoated plates (Fluka, Steinheim, Germany) using the following solvent systems: *n*-Hexane: ethyl acetate (8:2 and 7:3 v/v). The chromatograms were visualized under UV light (at 254 and 366 nm) and sprayed with *p*-anisaldehyde sulphuric acid spray reagent. Bruker NMR was used for ^1^H-NMR (400 MHz) and ^13^C-NMR (125 MHz) measurements. The NMR spectra were recorded in CD_3_OD and DMSO. Chemical shifts are given in *δ* (ppm) relative to internal standard TMS.

### Isolation of the compounds

The ethanolic extract (50 gm) was chromatographed on silica gel column (3.5 D x 25 cm L.). Elution of the column was done using solvents with increasing polarities starting with *n*-hexane-ethyl acetate (9.5:0.5 v/v) to (7:3 v/v), to afford 3 main compounds. ALUP (colorless microcrystalline powders, 255 mg), BA (colorless microcrystalline powder, 100 mg) and LUP (colorless microcrystalline powders, 500 mg).

### Cell culture

All the cell lines used in this study were obtained from Holding Company for Biological Products and Vaccines VACSERA, Egypt; MCF-7 cells (Human breast adenocarcinoma), HepG2 (human hepatocellular carcinoma), Caco-2 (colon carcinoma), A549 (human lung adenocarcinoma), PANC-1 (human pancreatic cancer), Vero cells (derived from normal kidney cells).

The RPMI 1640 medium (Lonza, Switzerland) was used to maintain all cell lines. The media also contained 10% fetal bovine serum (Gibco, USA), 1% penicillin, and 1% streptomycin (Sigma Aldrich, USA). In a humidified cell incubator with a 5% Carbon dioxide environment, the cells were kept at 37 °C. The study was examined and approved by the Ethics committee in the Faculty of Pharmacy-October University for Modern Sciences and Arts (MSA) with ethics approval number (BP2/Ec2/2021PD).

### MTT assay

In a 96-well tissue culture plate, all cell lines were planted at a density of 1 × 10^5^ cells per ml (100 µl per well), and they were then allowed to grow for 24 h at 37 °C to produce a full monolayer sheet. The growth medium from the 96 well micro titer was drained once the cells had formed a merged sheet, then replaced with different concentrations of compounds (ALUP, BA, LUP & Doxorubicin) (µg /ml) for 48 h, where the compounds were priorly dissolved in RPMI before adding to culture medium, the plate was incubated at 37 °C. MTT solution was prepared (5 mg/ml in Phosphate buffer saline (PBS)) (BIO BASIC, Canada), added to each well and incubated for 4 h, then the media was removed. To dissolve the formazan crystals, 200 µl of DMSO (dimethylsulfoxide) (Sigma Aldrich, USA) was added to each well. A microplate reader was then used to measure the formazan product’s optical density at 560 nm (Mindray, MR-96 A, China). The average of the three separate experiments was used to calculate the results.

The percentage of viability was calculated by using the following formula:

$$\%\;\mathrm{Of}\;\mathrm{cell}\;\mathrm{viability}\:=\:\mathrm{OD}\;\mathrm{of}\;\mathrm{Sample}/\mathrm{OD}\;\mathrm{of}\;\mathrm{Control}\;\times\;100$$

OD: optical density.

### Cell cycle analysis and apoptosis assay

A549 cells were developed and attached to the well walls during night. Following a 24-hour incubation period, A549 cells were treated with IC_50_ of ALUP, BA, and LUP for 48 h before being rinsed three times with ice-cold PBS. Both the treated and untreated cells were collected and harvested. After that, cells were reconstituted in 500 µl of binding buffer. In the dark for five minutes, 5 µl of each of annexin V-fluorescein isothiocyanate (FITC) (Biovision, USA) and propidium iodide (PI) (abcam, UK) were added. Using flow cytometry, cell cycle distribution was measured (BD FACSCalibur, India).

To distinguish between cell phases, we used a double staining kit (BioVision Research Products, USA) to stain the cells at room temperature with Annexin V- FITC and PI. Following the manufacturer’s instructions, apoptotic cells’ surface exposure to phosphatidylserine was measured using an Annexin V-FITC apoptosis detection kit (Biovision, USA).

### RNA extraction

A549 cells were allowed 48 h of incubation with IC_50_ concentrations of µM of ALUP, BA, and LUP. Following the manufacturer’s instructions, total RNA samples were extracted using the RNeasy mini kit (Qiagen, GmbH Germany).

### Quantitative polymerase chain reaction (qPCR)

*Bax, CASP-8, CD95 (cluster of differentiation 95), Bcl-2, KRAS (Kirsten rat sarcoma viral oncogene*), *VEGF, Cyclin D1*, and the housekeeping gene *β-actin* were among the target genes for which highly purified primers were created (Sigma, USA) (Supplementary Table S1). On a Rotor-Gene Q real-time PCR cycler, cDNA synthesis and PCR amplification were performed using the iScript TM One-Step RT-PCR Kit with SYBR® Green (Bio-Rad Laboratories, Canada) in accordance with the manufacturer’s instructions.

To determine the relative expression of the examined genes’ mRNA, real-time data were used. In order to enable the normalization of all data, the β-actin gene was used. Finally, values were reported as fold change using the equation: 2^ ΔΔCT [18].

Let-7 miRNA & miRNA-21 expression was analyzed using qRT-PCR using the TaqMan micro-RNA assay (Applied Biosystems Inc.). Small nuclear RNAU6 (RNU6B; Applied Biosystems Inc.) was used for normalization.

### Statistical analysis

The Graph Pad Prism 6 program was used to evaluate the data. Values were expressed as the mean ± SD of the triplicates of each experiment. For normally distributed quantitative variables, a two-way ANOVA with multiple comparisons post hoc test was utilized. A statistically significant *P* value of less than 0.05 was acceptable.

## Results

### Identification of the isolated compounds

**Fig. 1:**
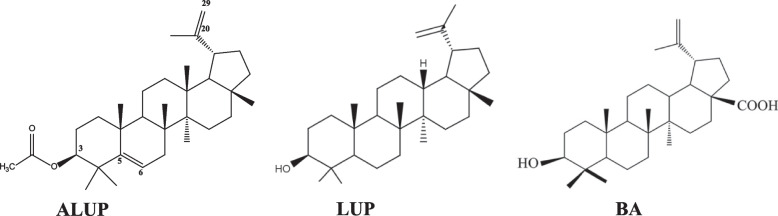
Structures of the isolated compounds, ALUP: acetoxy-lup-5(6), 20(29)-diene, LUP: lupeol and BA: betulinic acid

The three isolated compounds were identified as acetoxy lup-5(6), 20(29)-diene (ALUP), betulinic acid (BA) and lupeol (LUP). ^1^ H and ^13^ C NMR data and the structure of the three isolated compounds are displayed in Table 1 and Fig. 1.

**Table 1:** ^1^HNMR and ^13^CNMR data of the isolated compound (ALUP, BA and LUP) (J in HZ)

No. of carbon	Compound ALUP		Compound BA		Compound LUP	
	δ_H_	δ_C_	δ_H_	δ_C_	δ_H_	δ_C_
1	2.25 (m), 3.13 (m)	36.3	0.84 (m), 1.64 (m)	38.8		38.7
2	1.81, 2.14 (m)	27.0	1.52, 1.64 (m)	27.3		27.4
3	4.46 (1 H,dd,J = 5.3;4.1)	80.9	3.19 (1 H,dd, J = 4.9,5.3)	79.03	3.23 (1 H,m)	79.0
4	-	37.7	-	38.7	-	38.8
5	-	139.8	-	55.3	-	55.3
6	5.26 (1 H,br.s)	118.8	1.42(m), 1.55(m)	18.2	1.39(m), 1.55(m)	18.3
7	1.37 (m), 1.55(m)	34.5	1.35(m), 1.40(m)	34.3	1.32(m), 1.40(m)	34.6
8	-	42.8	-	40.7	-	40.8
9	1.86 (br.s)	55.3	1.25 (br.s)	50.5	1.29 (br.s)	50.4
10	-	37.7	-	37.2	-	37.1
11	2.01 (m)	22.5	1.37 (m)	20.8	1.32 (m)	21.1
12	1.62, 2.44 (m)	23.7	1.04,1.68 (m)	25.5	1.03,1.66 (m)	25.1
13	2.93 (m)	32.5	2.20 (m)	38.4	2.23 (m)	38.0
14	-	46.7	-	42.4	-	42.8
15	1.21 (m)	32.4	1.14(m)	30.5	1.16 (m)	27.6
16	1.51, 2.62 (m)	34.1	1.27(m)	32.1	1.28 (m)	35.5
17	-	55.3	-	56.3	-	43.0
18	1.78 (m)	50.3	1.61(m)	49.2	1.62 (m)	47.9
19	3.52 (m)	39.2	3.00 (m)	46.9	2.36 (m)	48.3
20	-	150.8	-	150.4		150.9
21	1.9, 2.24 (m)	31.0	1.99, 1.44(m)	37.0		29.9
22	1.54, 2.25(m)	33.3	1.55, 1.96(m)	29.6		40.0
23	1.40 (3 H,s)	29.8	0.87 (3 H,s)	27.5	0.78 (3 H, s)	28.0
24	0.95 (3 H,s)	22.6	0.77 (3 H,s)	15.3	0.81 (3 H, s)	15.3
25	1.29 (3 H,s)	16.5	1.27 (3 H,s)	16.0	0.85 (3 H, s)	16.1
26	1.06 (3 H,s)	17.7	0.98 (3 H,s)	16.1	0.96 (3 H, s)	15.9
27	1.04 (3 H,s)	14.7	0.95 (3 H,s)	14.6	0.99 (3 H, s)	14.5
28	0.99 (3 H,s)	14.5	-	181.1	1.05 (3 H, s)	18.0
29	4.58, 4.69 (br.s)	109.3	4.62,4.75 (br.s)	109.6	4.59 (1 H, br.s, H-29a) 4.70 (1 H, br.s, H-29b)	109.3
30	1.71 (3 H,s)	19.3	1.71 (3 H,s)	19.3	1.70 (3 H,s)	19.3
CH3-C = O	2.05 (3 H,s)	20.9				
CH3-C = O		170.9				

Acetoxy-lup-5(6), 20(29)-diene (ALUP) was isolated as a colorless microcrystalline powder from n-hexane-ethyl acetate (9.5:0.5 v/v) eluate. It responded positively to Liebermann-Burchard test for triterpenes. The ^1^H-NMR data (Table 1) of ALUP showed a one-proton broad singlet at δ 5.26 ppm assigned to the vinyl proton H-6. Another two one-proton broad singlets at δ 4.58 and 4.69 ppm accounted for the unsaturated methylene protons H2-29 [19]. A doublet at δ 4.46 ppm with J = 5.3 and 4.1 Hz assigned for H-3. The downfield shift of H-3 (δ_H_ 4.46 ppm and δ_C_ 80.9 ppm indicates acetylation of the 3-OH group. The acetyl group was detected at δ_H_ 2.05 ppm and δ_C_ 20.9 ppm for the CH_3_-C = O, and the carbonyl carbon δ_C_ 170.9 ppm. The acetylation of 3-OH was further confirmed from the correlations in HSQC and HMBC between the C = O (at *δ*C 170.9 ppm) and H-3 at *δ*_H_ 4.46 ppm. A singlet at δ 1.71 ppm was assigned to the C-30 methyl attached to the 20-vinylic carbon. Six singlets were detected at 1.40, 0.95, 1.29, 1.06, 1.04, and 0.99 assigned to the methyl groups at C-23, C-24, C-25, C-26, C-27, and C-28, respectively. The ^13^C-NMR data (Table 1) of ALUP showed 32 carbon signals. The vinylic carbons were identified and confirmed with HMBC correlations at δ_C_ 139.8, 118.8, 150.8, and 109.3 assigned to C-5, C-6, C-20, and C-29, respectively. The ^13^C-NMR data were comparable to the reported for the lupene-type triterpene [20]. Based on the above data, ALUP was identified as Acetoxy-lup-5(6), 20(29)-diene which is a new natural product.

Compound LUP was obtained as a colorless microcrystalline powder at n-hexane-ethyl acetate (9:1 v/v) and was identified as lupeol by comparing its ^1^H-NMR and ^13^C-NMR data with the published data [21]. The third compound BA was obtained by using n-hexane-ethyl acetate (8:2 v/v) where its ^1^H-NMR and ^13^C-NMR data were identical to those reported for betulinic acid [22].

### MTT assay

The cytotoxic effects of ALUP, BA, and LUP were evaluated on Vero, MCF-7, A549, Caco-2, HapG-2 and PANC-1 using MTT assay. The cell viability and toxicity were evaluated after 48 h (Table 2 & Supplementary Tables S2 to S7). IC_50_ values for ALUP, BA, and LUP against different cell lines were calculated showing the lowest IC_50_ for the three compounds against A549: (0.805 µM, 0.836 µM, 0.808 µM respectively)

**Table 2:** IC50 of ALUP, BA, LUP, and Doxorubicin against different cancer cell lines

Cell lines	IC50 Doxorubicin (µg/ml)	IC50 ALUP (µg/ml)	IC50 BA (µg/ml)	IC50 LUP (µg/ml)
**Vero**	25.83	824.9	4543.3	3645.5
**MCF-7**	94.04	594.4	569.7	982.09
**A549**	76.2	376.1	381.9	344.8
**Caco-2**	95.47	813.9	375.1	384.5
**HepG2**	98.55	371.9	471.1	606.6
**PANC-1**	89.31	668.5	291.4	402.6

### Flow cytometry analysis using annexin V binding and PI staining

**Fig. 2:**
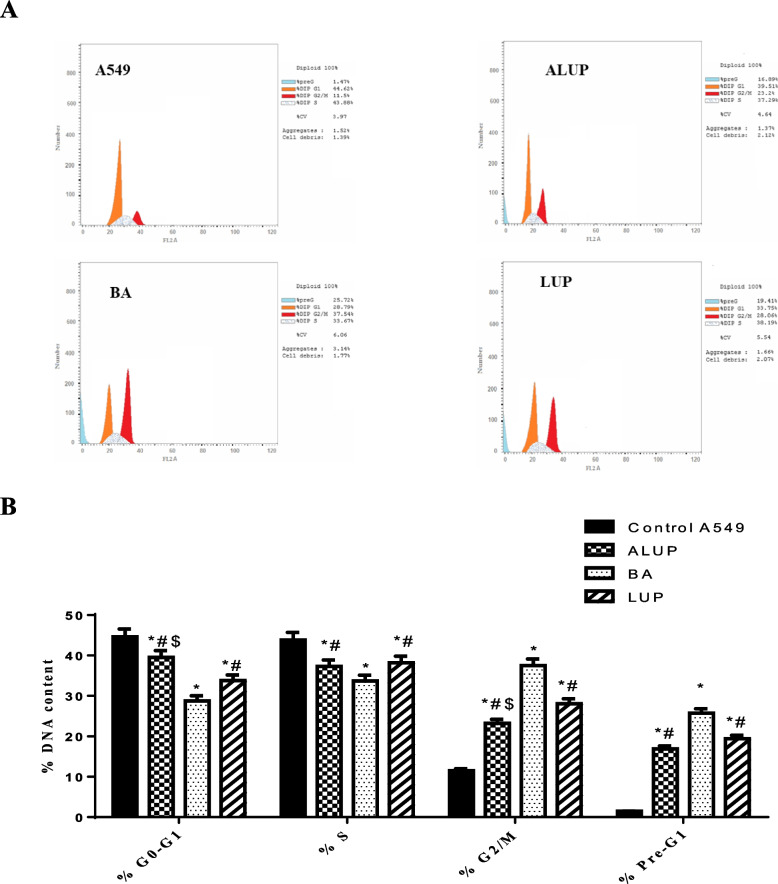
Cell cycle analysis of A549 (untreated) and treated with IC50 ALUP, BA, LUP for 48 h ALUP, BA, LUP (0.805 µM, 0.836 µM, 0.808 µM respectively): **A **Histogram showing the percentage of cell population in each phase of cell cycle analysis. **B **Bar chart showing the percentage of cell population in each phase of the cell cycle analysis. * Significance from A549 cells (untreated) at *P* < 0.0001, # Significance from BA treatment at *P* < 0.001, $ Significance from LUP treatment at *P* < 0.001

Table 3 and  Fig. 2 showed that the cell cycle arrest was induced at the G2/M phase after the treatment with IC_50_ ALUP, BA, and LUP (0.805 µM, 0.836 µM, 0.808 µM, respectively) with an increase in the apoptosis.

**Table 3:** Percentage distribution of cell cycle stages in A549 cells treated with ALUP, BA, LUP

	%G0-G1	%S	%G2/M	%Pre-G1
**Control A549**	44.62 ± 1.91	43.88 ± 1.88	11.5 ± 0.49	1.47 ± 0.06
**ALUP**	39.51 ± 1.69*#$	37.29 ± 1.6*#	23.2 ± 0.99*#$	16.89 ± 0.72*#
**BA**	28.79 ± 1.23*	33.67 ± 1.44*	37.54 ± 1.61*	25.72 ± 1.1*
**LUP**	33.75 ± 1.45*#	38.19 ± 1.64*#	28.06 ± 1.2*#	19.41 ± 0.83*#

* Significance from A549 cells (untreated) at *P* < 0.0001

# Significance from BA treatment at *P* < 0.001

$ Significance from LUP treatment at *P* < 0.001

In the pre-G1 phase, the percentage of A549 cells was statistically raised from 1.47 to 25.72% (*P* < 0.0001), also in the G2/M phase it was statistically raised from 11.5 to 37.54% (*P* < 0.0001), meanwhile it was decreased in G0/G1 phase from 44.62 to 28.79% in the untreated and treated cells with BA.

**Fig. 3:**
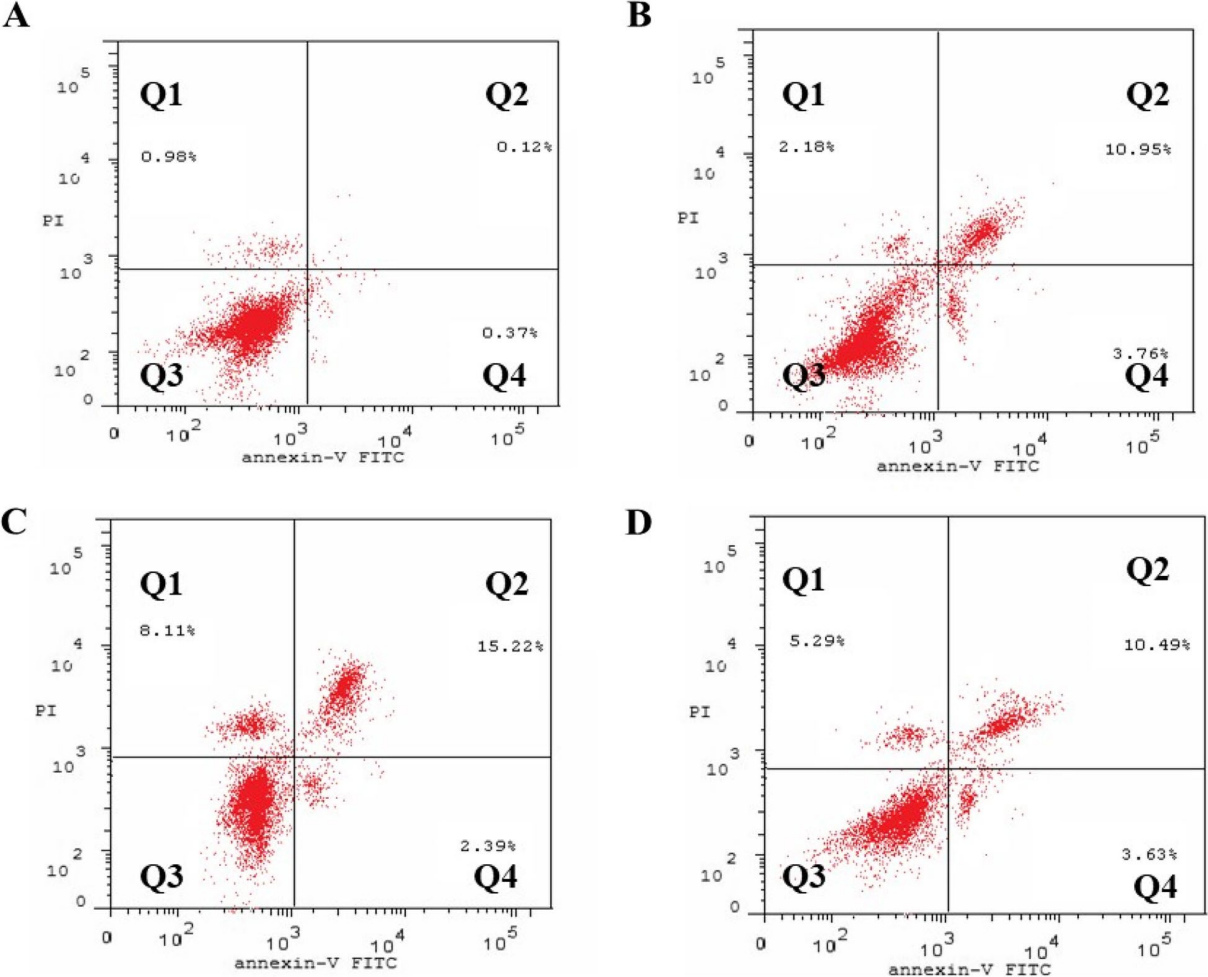
Dot plot representing four quadrant images recorded by flow cytometry analysis for cells stained by Annexin V-FITC and propidium iodide. Q1: shows necrotic cells, Q2: shows later period apoptotic cells, Q3: shows normal cells, Q4: shows early apoptotic cells. **A** Control cells, **B** ALUP, **C** BA, **D** LUP

After ALUP, BA, and LUP treatment, cells were aggregated at the G2/M phase, thus we tried to measure the number of cells in each stage of apoptosis. Early apoptotic cells (annexin V-positive/PI-negative) and late apoptotic cells were distinguished using Annexin V-FITC/PI double staining (annexin V-positive and PI-positive). The concentration of cells in the late apoptotic stage was increased after the treatment with ALUP, BA and LUP with the highest concentration in BA treated cells (Table 4 and Fig. 3).

**Table 4:** Concentration of cells (ug/ml) detected at different types of apoptosis induced in A549 cells following treatment with ALUP, BA, and LUP.

	Total	Early	Late	Necrosis
**Control A549**	1.47 ± 0.08	0.37 ± 0.02	0.12 ± 0.01	0.98 ± 0.05
**ALUP**	16.89 ± 0.91*#$	3.76 ± 0.2*#	10.95 ± 0.59*#	2.18 ± 0.12#$
**BA**	25.72 ± 1.38*	2.39 ± 0.13*	15.22 ± 0.82*	8.11 ± 0.43*
**LUP**	19.41 ± 1.04*#	3.63 ± 0.19*	10.49 ± 0.56*#	5.29 ± 0.28*#

* Significance from A549 cells (untreated) at *P* < 0.001

# Significance from BA treatment at *P* < 0.05

$ Significance from LUP treatment at *P* < 0.001

### Regulation of Let-7 miRNA and miR-21 expression by ALUP, BA & LUP

**Fig. 4:**
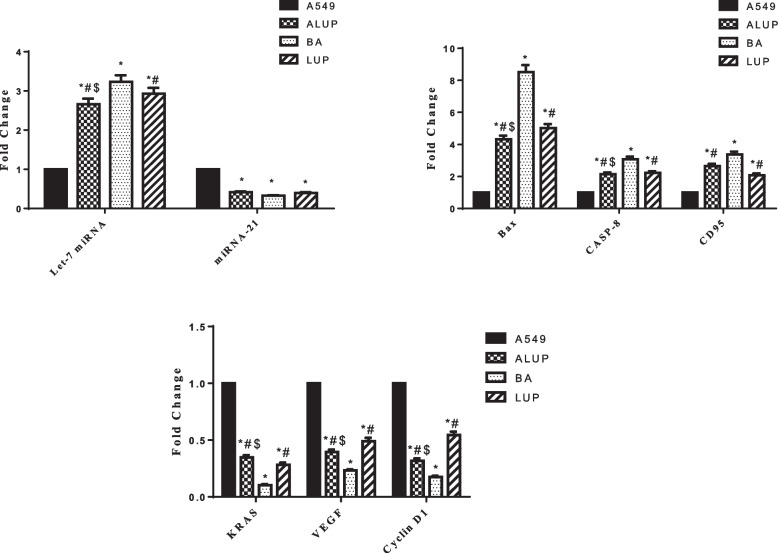
Real-time PCR analysis data depicting the relative normalized expression of let-7 miRNA, miRNA-21, Bax, CASP-8, CD95, KRAS, VEGF and Cyclin D1 after A549 treatment with ALUP, BA and LUP. *P*-values represented on the graph reflect the statistical significance of various treatments in comparison to untreated cells. The relative expression was calculate based on 2 − ΔΔCt method. * Significance from A549 cells (untreated) at *P* < 0.0001, # Significance from BA treatment at *P* < 0.001, $ Significance from LUP treatment at *P* < 0.01

RT-PCR results revealed that BA treatment significantly raised the expression of let-7 miRNA (3.23 ± 0.17) in A549 cells compared to other treatments at *P* < 0.001. While the expression of miRNA-21 level was statistically lowered in all treated cells when compared to untreated cells, but there was no significant difference between all treated cells as shown in Table 5 and Fig. 4.

**Table 5:** Effect of ALUP, BA, and LUP on the expression level of different markers in the A549 cell line

	Let-7	miRNA-21	Bax	CASP-8	CD95	Bcl-2
**Control A549**	1	1	1	1	1	1
**ALUP**	2.66 ± 0.14*#$	0.41 ± 0.02*	4.33 ± 0.22*#$	2.14 ± 0.11*#	2.65 ± 0.14*#$	0.401 ± 0.02*
**BA**	3.23 ± 0.17*	0.33 ± 0.01*	8.52 ± 0.43*	3.08 ± 0.16*	3.38 ± 0.17*	0.26 ± 0.01*
**LUP**	2.93 ± 0.15*#	0.39 ± 0.02*	5.02 ± 0.26*#	2.22 ± 0.11*#	2.08 ± 0.11*#	0.35 ± 0.02*

* Significant from A549 cells (untreated) at *P* < 0.0001

# Significant from BA treatment at *P* < 0.001

$ Significance from LUP treatment at P < 0.01

### Regulation of bax, Caspase-8, CD95 and Bcl-2 mRNA expression by ALUP, BA & LUP

Table 5 and Fig. 4 showed that ALUP, BA & LUP treatments significantly raised the expression levels of Bax, CASP-8 & CD95 in comparison to untreated cells at *P* < 0.0001, meanwhile BA treatment significantly increased their expression compared to other treatments at *P* < 0.001. All treated cells had lower levels of anti-apoptotic Bcl-2 mRNA than untreated cells, but there was no statistically significant difference between the various treatments.

### Regulation of KRAS, VEGF, and cyclin D1 mRNA expression by ALUP, BA & LUP

The mRNA expression levels of KRAS, VEGF, and Cyclin D1 were significantly lowered in all treated cells in comparison to untreated cells at *P* < 0.0001, BA treatment showed a prominent effect in decreasing their expression levels compared to other treated groups at *P* < 0.001 (Table 6 and Fig. 4).

**Table 6:** Effect of ALUP, BA, and LUP on KRAS, VEGF, and Cyclin D1 expression in the A549 cell line

	KRAS	VEGF	Cyclin D1
**Control A549**	1	1	1
**ALUP**	0.347 ± 0.02*#$	0.396 ± 0.02*#$	0.318 ± 0.02*#$
**BA**	0.102 ± 0.01*	0.232 ± 0.01*	0.174 ± 0.01*
**LUP**	0.282 ± 0.02*#	0.491 ± 0.03*#	0.544 ± 0.03*#

* Significant from A549 cells (untreated) at *P* < 0.0001

# Significant from BA treatment at *P* < 0.001

$ Significance from LUP treatment at P < 0.01

## Discussion

Natural products represent a treasure in drug discovery for the treatment of various diseases, it was reported that 50% of the approved drugs since 1946 originate from natural origin [23]. Lung cancer is one of the leading causes of death worldwide, though it was found that dietary supplements such as tomatoes, turmeric, grapes, ginger, and pomegranates, as well as their constituents, may play a crucial role against this type of cancer, through induction of apoptosis, inhibition of proliferation or increasing cell sensitivity to anticancer agents [24]. Thus, we aimed in our study to isolate the major triterpenes from the ethanolic extract of *T. capitatus* in order to investigate their underlying mechanism in inducing apoptosis via the regulation of Let-7 miRNA/Cyclin D1/VEGF cascade in non-small cell lung carcinoma (NSCLC).

Extraction and purification of the ethanolic extract of the aerial parts of *T. capitatus* yielded three compounds: ALUP, LUP, and BA. Acetoxy-lup-5(6), 20(29)-diene (ALUP), lupeol (LUP) and betulinic acid (BA).

We evaluated the viability of the cells using MTT assay, cell cycle analysis by flow cytometry, and mRNA expression of Let-7-miRNA, miRNA-21, Bax, CASP-8, CD-95, KRAS, VEGF, and cyclin D1 by RT-qPCR in order to better understand the mechanism of action of three isolated compounds.

Our results demonstrated that the cytotoxic effects of the three isolated compounds ALUP, BA, and LUP on Vero, MCF-7, A549, Caco-2, HepG-2, and PANC-1 using MTT assay showed that the three compounds reduced the viability of A549 human lung cancer cell line with IC_50_ values of 0.805 µM, 0.836 µM, 0.808 µM, respectively. This comes in agreement with Pisha et al.,1995 in which betulinic acid demonstrated tumor-related cytotoxicity toward melanoma cell lines [25]. Several reports have shown that treatment with betulinic acid has a prominent effect on multiple human cancer cell lines one of them is lung carcinoma [26]. Lupeol has also been demonstrated to be beneficial against lung cancer (specifically, A427 cancer cells and healthy MRC-5 cells). The MTT assay is used to verify the observation of growth inhibition of lung cancer cells [27]. In another study performed by Wróblewska-Łuczka et al., 2022, different doses of BA were administered to four melanoma cell lines (A375, SK-MEL28, FM55P, and FM55M2) as well as healthy human keratinocytes (HaCaT) resulting in decreased viability of the melanoma cells in a concentration-dependent manner [28].

Our study showed that treatment of A549 cells with ALUP, BA, and LUP at the IC_50_ for 48 h induced cell cycle arrest at the G2/M phase. Furthermore, the isolated compounds induced apoptosis explicated by the high proportion of cells that accumulated in the late apoptotic stage with the highest concentration in BA-treated cells. These results agree with those of Zhan et al., 2018 who reported that treating paclitaxel-resistant lung cancer cells (H460) with betulinic acid induced a G2/M phase cell cycle arrest.

Apoptosis (programmed cell death) may be observed in both physiologically and pathologically mediated through two major pathways intrinsic and extrinsic; where the intrinsic pathway is specifically linked to mitochondrial outer membrane permeabilization and is activated by DNA damage, the mitochondrial pathway is based mainly on the activity of the Bcl-2 family proteins [29], and the extrinsic pathway is dependent on receptor-ligand ligation as CD95-L to its receptor CD95 leading to several cascades that proceed to the activation of caspase-8 and caspase-10 [30]. Where the activated caspase-8 can directly stimulate caspase-3 activity [31].

Moving to our results we found that the treatment of the A549 human lung cancer cell line with ALUP, BA, and LUP significantly increased the mRNA expression of the apoptotic promotors Bax (the pro-apoptotic), CASP-8, and CD95, with BA treatment showing the most pronounced effect.

The upregulation of Bcl-2 gene expression levels can promote the survival of the cancer cells, therefore the inhibition of the anti-apoptotic/pro-survival members of the Bcl-2 family of proteins is an attractive approach for combating cancer [32]. Treatment of A549 with ALUP, BA, and LUP compounds caused downregulation of the anti-apoptotic Bcl-2 mRNA and this contributes to the apoptosis-promoting activity of our compounds.

It was evidenced that the let-7 miRNA family is involved in the proliferation and differentiation of cell development. Additionally, the deregulation of Let-7 miRNA has been demonstrated to be a feature of numerous cancers specifically lung cancer, Let-7a miRNA may target a number of genes, including RAS, Myc, Hmga2, and Cyclin D1 [33].

Cyclin D1 is a necessary protein for the cell cycle to continue through the G1 phase, where its over-expression has been linked to early cancer onset and tumor development and could promote oncogenesis by boosting anchorage-independent growth and angiogenesis via VEGF production [34]. Also, it plays a role as a prognostic factor in many human cancers [35].

Vascular endothelial growth factor (VEGF) is a proangiogenic factor that is upregulated in various tumors and plays a pivotal role in the development and progression of malignancies; where it promotes endothelial cell survival, proliferation, and migration, loosening intercellular connections to increase vascular permeability, and recruitment of endothelial progenitor and other cells [36].

Our study revealed that treatment of A549 human lung cancer cell line with ALUP, BA and LUP significantly increased the expression of let-7 miRNA and reduced the expression of Cyclin D1, KRAS and VEGF levels. where BA treatment showed the most prominent effect. These results are in alignment with *Tian et al.*, 2017 where they found that the activation of the p15/cyclin D1 pathway greatly promotes NSCLC carcinogenesis [37], another supportive study showed that the anti-proliferative effect of betulinic acid treatment on various cancer cell lines via decreasing the expression levels of Bcl-2, cyclin D1 genes [38].

Since, miRNA-21 is frequently up-regulated in various cancer cells such as lung, breast, and colon cancers, it is believed that it is an oncogene that plays a key role in resisting programmed cell death [39]. Our study revealed that treatment of A549 human cells with ALUP, BA, and LUP significantly decreased the expression of miRNA-21 level in the treated cells compared to the untreated cells. In agreement with our results, in a study carried out by *Zhou et al., 2018*, where miR-21 was over-expressed in A549 cells compared to normal cells, also the inhibition of miR-21 induced the apoptosis in A549 cells via the caspase-dependent pathway, which reflected on the reduced expression level of Bcl-2 and increased expression level of Bax in A549 cells [40]. This is the first report to demonstrate the anti-cancer activity of newly isolated compound acetoxy-lup-5(6), 20(29)-diene (ALUP) in reducing the proliferation and differentiation on A549 human non-small cell lung cancer cell line via inhibition of mitotic phase, increased apoptosis, overexpression of Let-7-miRNA, Bax, CASP-8, CD-95 and down expression of miRNA-21, Bcl-2, KRAS, VEGF, and cyclin D1.

## Conclusion

A novel compound identified as Acetoxy-lup-5(6), 20(29)-diene was isolated from the aerial parts of *Thymus capitatus*, together with the known triterpenes lupeol and betulinic acid. The three isolated compounds inhibited the proliferation of human lung cancer cell line A549 and induced cell cycle arrest followed by the induction of apoptosis through its effect on the expression of various apoptosis promotors and miRNA expression. Finally by targeting the Let-7 miRNA/Cyclin D1/VEGF cascade, acetoxy-lup-5(6), 20(29)-diene could be a potential therapeutic agent for lung cancer. These findings may support the value of carrying out further preclinical and clinical trials.

### Supplementary Information

**Additional file 1:** **Table S1. **Primer list for real-time polymerase chain reaction. **Table S2.** Cytotoxicity evaluation of ALUP, BA, and LUP at various concentrations against Vero cell line. **Table S3.** Cytotoxicity evaluation of ALUP, BA, and LUP at various concentrations against MCF-7 cancer cell line. **Table S4.** Cytotoxicity evaluation of ALUP, BA, and LUP at various concentrations against A549 cancer cell line. **Table S5.** Cytotoxicity evaluation of ALUP, BA, and LUP at various concentrations against Caco-2 cancer cell line. **Table S6.** Cytotoxicity evaluation of ALUP, BA, and LUP at various concentrations against HepG2 cancer cell line. **Table S7.** Cytotoxicity evaluation of ALUP, BA, and LUP at various concentrations against PANC-1 cancer cell line.

## Data Availability

The datasets used and/or analyzed during the current study available from the corresponding author on reasonable request.

## Declarations

- The aerial parts of *Thymus capitatus* (L.) Hoffmanns. & Link were collected from North Coast, Egypt in the Spring of 2019 in accordance with national and international guidelines. Egypt does not apply any restrictions on plant collection for nonprofit or research use; especially that our plant is not endangered or at risk of extinction, however verbal permission was taken from the Alexandrian Governorate.

The plant was authenticated in the Herbarium of the Faculty of Science, Cairo University. A voucher specimen (6-05-2019) was kept in the herbarium of the Pharmacognosy Department, Faculty of Pharmacy, Cairo University, Cairo, Egypt, with a deposition number (TC-00107).
