# Supplementary material for: A novel lupene derivative from Thymus capitatus possesses an apoptosis-inducing effect via Let-7 miRNA/Cyclin D1/VEGF cascade in the A549 cell line
Source: BMC Complement Med Ther. 2023 Oct 16;23:365. doi: 10.1186/s12906-023-04201-7 (PMC10577955; doi:10.1186/s12906-023-04201-7)
Supplement: Supplementary file 1 — Additional file 1: Table S1. Primer list for real-time polymerase chain reaction. Table S2. Cytotoxicity evaluation of ALUP, BA, and LUP at various concentrations against Vero cell line. Table S3. Cytotoxicity evaluation of ALUP, BA, and LUP at various concentrations against MCF-7 cancer cell line. Table S4. Cytotoxicity evaluation of ALUP, BA, and LUP at various concentrations against A549 cancer cell line. Table S5. Cytotoxicity evaluation of ALUP, BA, and LUP at various concentrations against Caco-2 cancer cell line. Table S6. Cytotoxicity evaluation of ALUP, BA, and LUP at various concentrations against HepG2 cancer cell line. Table S7. Cytotoxicity evaluation of ALUP, BA, and LUP at various concentrations against PANC-1 cancer cell line. [file 12906_2023_4201_MOESM1_ESM.docx]

**Table S1: Primer list for real-time polymerase chain reaction.**

| Bax | Forward | 5’-ATGTTTTCTGACGGCAACTTC-3’ |
| --- | --- | --- |
|  | Reverse | 5’-AGTCCAATGTCCAGCCCAT-3’ |
| Bcl-2 | Forward | 5’-ATGTGTGTGGAGACCGTCAA-3’ |
|  | Reverse | 5’-GCCGTACAGTTCCACAAAGG-3’ |
| VEGF | Forward | 5′-TGCAGATTATGCGGATCAAACC-3' |
|  | Reverse | 5'-TGCATTCACATTTGTTGTGCTGTAG-3' |
| Caspase-8 | Forward | 5′-AATGTTGGAGGAAAGCAATC-3′ |
|  | Reverse | 5′-CATAGTCGTTGATTATCTTCAGC-3′ |
| KRAS | Forward | 5'-GCCTGCTGAAAATGACTGAATATA-3' |
|  | Reverse | 5'-TTAGCTGTATCGTCAAGGCACTC-3' |
| Cyclin D1 | Forward | 5’-AGACCTGCGCGCCCTCGGTG-3’ |
|  | Reverse | 5’-GTAGTAGGACAGGAAGTTGTTC-3’ |
| CD 95 | Forward | 5'-TGAAGGACATGGCTTAGAAGTG-3' |
|  | Reverse | 5'-GGTGCAAGGGTCACAGTGTT-3' |
| β-actin | Forward | 5′-TGACGTGGACATCCGCAAAG-3′ |
|  | Reverse | 5′-CTGGAAGGTGGACAGCGAGG-3′ |
| miRNA-21 | Forward | 5′-GGGTAGCTTATCAGACTGAT-3′ |
|  | Reverse | 5′-CAGTGCGTGTCGTGGAGT-3′ |
| let-7 miRNA | Forward | 5′-GCCGCTGAGGTAGTAGGTTGTA-3′ |
|  | Reverse | 5′- GTGCAGGGTCCGAGGT-3′ |
| U6 | Forward | 5’-CCTGCTTCGGCAGCACA-3’ |
|  | Reverse | 5’-TGGAACGCTTCACGAA-3’ |

**Table S2. Cytotoxicity evaluation of ALUP, BA, and LUP at various concentrations against Vero cell line**

| ID | Conc. (ug/ml) | Mean O.D | Viability % | Toxicity % | IC50 (µg/ml) | IC50 (µM) |
| --- | --- | --- | --- | --- | --- | --- |
| Vero | --- | 0.282 ± 0.007 | 100 | 0 |  |  |
| ALUP | 1000 | 0.088 ± 0.004 | 31.3 | 68.7 | 824.9 | 1.76 |
|  | 500 | 0.24 ± 0.005 | 87.1 | 12.9 |  |  |
|  | 250 | 0.28 ± 0.002 | 100 | 0 |  |  |
|  | 125 | 0.28 ± 0.006 | 100 | 0 |  |  |
|  | 62.5 | 0.28 ± 0.001 | 100 | 0 |  |  |
|  | 31.25 | 0.28 ± 0.005 | 100 | 0 |  |  |
| BA | 5000 | 0.134 ± 0.004 | 40 | 60 | 4543.3 | 9.95 |
|  | 2500 | 0.307 ± 0.006 | 91.8 | 8.1 |  |  |
|  | 1250 | 0.33 ± 0.005 | 99.5 | 0.49 |  |  |
|  | 625 | 0.33 ± 0.007 | 99.3 | 0.69 |  |  |
|  | 312.5 | 0.336 ± 0.003 | 100 | 0 |  |  |
|  | 156.25 | 0.33 ± 0.007 | 99.5 | 0.49 |  |  |
| LUP | 5000 | 0.093 ± 0.004 | 27.9 | 72.03 | 3645.5 | 8.54 |
|  | 2500 | 0.021 ± 0.007 | 63.78 | 36.2 |  |  |
|  | 1250 | 0.32 ± 0.006 | 97.7 | 2.3 |  |  |
|  | 625 | 0.33 ± 0.002 | 99.01 | 0.99 |  |  |
|  | 312.5 | 0.33 ± 0.006 | 99.8 | 0.19 |  |  |
|  | 156.25 | 0.33 ± 0.004 | 100 | 0 |  |  |
| Doxorubicin | 1000 | 0.022± 0.005 | 8.76 | 91.2 | 25.83 |  |
|  | 500 | 0.024 ± 0.006 | 9.82 | 90.1 |  |  |
|  | 250 | 0.031 ± 0.003 | 12.6 | 87.4 |  |  |
|  | 125 | 0.046 ± 0.002 | 18.4 | 81.5 |  |  |
|  | 62.5 | 0.055 ± 0.006 | 22.04 | 77.9 |  |  |
|  | 31.25 | 0.10 ± 0.002 | 40.9 | 59.09 |  |  |
|  | 15.62 | 0.158 ± 0.005 | 63.1 | 36.9 |  |  |

**Table S3. Cytotoxicity evaluation of ALUP, BA, and LUP at various concentrations against MCF-7 cancer cell line**

| ID | Conc. (ug/ml) | Mean O.D | Viability % | Toxicity % | IC50 (µg/ml) | IC50 (µM) |
| --- | --- | --- | --- | --- | --- | --- |
| MCF-7 | --- | 0.312 ± 0.009 | 100 | 0 |  |  |
| ALUP | 1000 | 0.048 ± 0.004 | 15.5 | 84.4 | 594.4 | 1.273 |
|  | 500 | 0.148 ± 0.001 | 47.5 | 52.45 |  |  |
|  | 250 | 0.279 ± 0.007 | 89.5 | 10.5 |  |  |
|  | 125 | 0.302 ± 0.003 | 96.9 | 3.1 |  |  |
|  | 62.5 | 0.305 ± 0.005 | 97.7 | 2.24 |  |  |
|  | 31.25 | 0.313 ± 0.002 | 100 | 0 |  |  |
| BA | 5000 | 0.019 ± 0.003 | 5.78 | 94.2 | 569.7 | 1.248 |
|  | 2500 | 0.019 ± 0.005 | 5.58 | 94.4 |  |  |
|  | 1250 | 0.062 ± 0.004 | 18.3 | 81.6 |  |  |
|  | 625 | 0.141 ± 0.009 | 41.4 | 58.5 |  |  |
|  | 312.5 | 0.294 ± 0.004 | 86.6 | 13.3 |  |  |
|  | 156.25 | 0.327 ± 0.004 | 96.1 | 3.82 |  |  |
| LUP | 5000 | 0.019 ± 0.005 | 5.58 | 94.4 | 982.09 | 2.303 |
|  | 2500 | 0.053 ± 0.003 | 15.5 | 84.4 |  |  |
|  | 1250 | 0.126 ± 0.005 | 37.1 | 62.8 |  |  |
|  | 625 | 0.218 ± 0.003 | 64.2 | 35.8 |  |  |
|  | 312.5 | 0.305 ± 0.002 | 89.9 | 10.1 |  |  |
|  | 156.25 | 0.327 ± 0.003 | 96.3 | 3.62 |  |  |
| Doxorubicin | 1000 | 0.019 ± 0.002 | 7.43 | 92.5 | 94.04 |  |
|  | 500 | 0.030 ± 0.003 | 11.7 | 88.2 |  |  |
|  | 250 | 0.068 ± 0.009 | 26.4 | 73.5 |  |  |
|  | 125 | 0.102 ± 0.008 | 39.3 | 60.6 |  |  |
|  | 62.5 | 0.144 ± 0.003 | 55.5 | 44.5 |  |  |
|  | 31.25 | 0.203 ± 0.003 | 78.2 | 21.8 |  |  |
|  | 15.62 | 0.255 ± 0.007 | 98.2 | 1.79 |  |  |

**Table S4. Cytotoxicity evaluation of ALUP, BA, and LUP at various concentrations against A549 cancer cell line**

| ID | Conc. (ug/ml) | Mean O.D | Viability % | Toxicity % | IC50 (µg/ml) | IC50 (µM) |
| --- | --- | --- | --- | --- | --- | --- |
| A549 | --- | 0.297 ± 0.005 | 100 | 0 |  |  |
| ALUP | 1000 | 0.027 ± 0.003 | 9.09 | 90.9 | 376.1 | 0.805 |
|  | 500 | 0.089 ± 0.005 | 29.9 | 70.03 |  |  |
|  | 250 | 0.202 ± 0.003 | 68.1 | 31.8 |  |  |
|  | 125 | 0.283 ± 0.004 | 95.3 | 4.60 |  |  |
|  | 62.5 | 0.296 ± 0.006 | 99.7 | 0.224 |  |  |
|  | 31.25 | 0.297 ± 0.006 | 100 | 0 |  |  |
| BA | 5000 | 0.021 ± 0.001 | 6.41 | 93.58 | 381.9 | 0.836 |
|  | 2500 | 0.022 ± 0.001 | 6.83 | 93.2 |  |  |
|  | 1250 | 0.028 ± 0.002 | 8.79 | 91.2 |  |  |
|  | 625 | 0.053 ± 0.007 | 16.45 | 83.5 |  |  |
|  | 312.5 | 0.165 ± 0.007 | 51.24 | 48.7 |  |  |
|  | 156.25 | 0.29 ± 0.005 | 90.16 | 9.83 |  |  |
| LUP | 5000 | 0.019 ± 0.001 | 5.90 | 94.1 | 344.8 | 0.808 |
|  | 2500 | 0.018 ± 0.001 | 5.79 | 94.2 |  |  |
|  | 1250 | 0.022 ± 0.001 | 6.83 | 93.2 |  |  |
|  | 625 | 0.034 ± 0.004 | 10.6 | 89.4 |  |  |
|  | 312.5 | 0.144 ± 0.005 | 44.7 | 55.3 |  |  |
|  | 156.25 | 0.27 ± 0.003 | 84.98 | 15.01 |  |  |
| Doxorubicin | 1000 | 0.029 ± 0.002 | 8.12 | 91.8 | 76.2 |  |
|  | 500 | 0.037 ± 0.001 | 10.45 | 89.5 |  |  |
|  | 250 | 0.055 ± 0.001 | 15.4 | 84.5 |  |  |
|  | 125 | 0.091 ± 0.003 | 25.5 | 74.5 |  |  |
|  | 62.5 | 0.156 ± 0.006 | 43.8 | 56.2 |  |  |
|  | 31.25 | 0.27 ± 0.003 | 75.6 | 24.4 |  |  |
|  | 15.62 | 0.346 ± 0.002 | 96.9 | 3.08 |  |  |

**Table S5. Cytotoxicity evaluation of ALUP, BA, and LUP at various concentrations against Caco-2 cancer cell line**

| ID | Conc. (ug/ml) | Mean O.D | Viability % | Toxicity % | IC50 (µg/ml) | IC50 (µM) |
| --- | --- | --- | --- | --- | --- | --- |
| Caco-2 | --- | 0.363 ± 0.004 | 100 | 0 |  |  |
| ALUP | 1000 | 0.11 ± 0.007 | 31.7 | 68.2 | 813.9 | 1.743 |
|  | 500 | 0.30 ± 0.002 | 82.7 | 17.3 |  |  |
|  | 250 | 0.361 ± 0.002 | 99.5 | 0.45 |  |  |
|  | 125 | 0.362 ± 0.004 | 99.9 | 0.09 |  |  |
|  | 62.5 | 0.364 ± 0.006 | 100 | 0 |  |  |
|  | 31.25 | 0.364 ± 0.002 | 100 | 0 |  |  |
| BA | 5000 | 0.018 ± 0.001 | 5.11 | 94.9 | 375.1 | 0.821 |
|  | 2500 | 0.02 ± 0.001 | 5.47 | 94.5 |  |  |
|  | 1250 | 0.021 ± 0.001 | 5.75 | 94.2 |  |  |
|  | 625 | 0.022 ± 0.001 | 6.11 | 93.8 |  |  |
|  | 312.5 | 0.19 ± 0.001 | 54.33 | 45.66 |  |  |
|  | 156.25 | 0.348 ± 0.004 | 95.4 | 4.56 |  |  |
| LUP | 5000 | 0.019 ± 0.001 | 5.29 | 94.7 | 384.5 | 0.901 |
|  | 2500 | 0.019 ± 0.001 | 5.29 | 94.7 |  |  |
|  | 1250 | 0.021 ± 0.001 | 5.75 | 94.2 |  |  |
|  | 625 | 0.022 ± 0.002 | 6.11 | 93.8 |  |  |
|  | 312.5 | 0.21 ± 0.002 | 57.1 | 42.9 |  |  |
|  | 156.25 | 0.35 ± 0.004 | 98.2 | 1.73 |  |  |
| Doxorubicin | 1000 | 0.032 ± 0.002 | 10.2 | 89.8 | 95.47 |  |
|  | 500 | 0.048 ± 0.003 | 15.1 | 84.9 |  |  |
|  | 250 | 0.091 ± 0.004 | 28.33 | 71.6 |  |  |
|  | 125 | 0.11 ± 0.001 | 36.45 | 63.5 |  |  |
|  | 62.5 | 0.19 ± 0.001 | 60.8 | 39.16 |  |  |
|  | 31.25 | 0.28 ± 0.003 | 87.5 | 12.5 |  |  |
|  | 15.62 | 0.32 ± 0.002 | 99.3 | 0.625 |  |  |

**Table S6. Cytotoxicity evaluation of ALUP, BA, and LUP at various concentrations against HepG2 cancer cell line**

| ID | Conc. (ug/ml) | Mean O.D | Viability % | Toxicity % | IC50 (µg/ml) | IC50 (µM) |
| --- | --- | --- | --- | --- | --- | --- |
| HepG2 | --- | 0.298 ± 0.004 | 100 | 0 |  |  |
| ALUP | 1000 | 0.027 ± 0.003 | 9.17 | 90.8 | 371.9 | 0.796 |
|  | 500 | 0.068 ± 0.003 | 22.9 | 77.1 |  |  |
|  | 250 | 0.238 ± 0.007 | 79.9 | 20.1 |  |  |
|  | 125 | 0.29 ± 0.004 | 98.1 | 1.90 |  |  |
|  | 62.5 | 0.299 ± 0.003 | 100 | 0 |  |  |
|  | 31.25 | 0.296 ± 0.007 | 99.4 | 0.56 |  |  |
| BA | 5000 | 0.019 ± 0.001 | 5.27 | 94.7 | 471.1 | 1.03 |
|  | 2500 | 0.02 ± 0.001 | 5.64 | 94.35 |  |  |
|  | 1250 | 0.023 ± 0.001 | 6.38 | 93.6 |  |  |
|  | 625 | 0.109 ± 0.003 | 30.27 | 69.7 |  |  |
|  | 312.5 | 0.237 ± 0.006 | 66.01 | 33.98 |  |  |
|  | 156.25 | 0.35 ± 0.002 | 99.07 | 0.92 |  |  |
| LUP | 5000 | 0.018 ± 0.001 | 5.18 | 94.8 | 606.6 | 1.42 |
|  | 2500 | 0.02 ± 0.001 | 5.74 | 94.2 |  |  |
|  | 1250 | 0.07 ± 0.007 | 20.9 | 79.1 |  |  |
|  | 625 | 0.167 ± 0.009 | 46.5 | 53.4 |  |  |
|  | 312.5 | 0.316 ± 0.006 | 87.8 | 12.2 |  |  |
|  | 156.25 | 0.35 ± 0.006 | 98.3 | 0.64 |  |  |
| Doxorubicin | 1000 | 0.03 ± 0.002 | 10.6 | 89.4 | 98.55 |  |
|  | 500 | 0.041 ± 0.003 | 14.5 | 85.5 |  |  |
|  | 250 | 0.051 ± 0.002 | 18.25 | 81.74 |  |  |
|  | 125 | 0.117 ± 0.006 | 41.46 | 58.5 |  |  |
|  | 62.5 | 0.155 ± 0.006 | 54.88 | 45.11 |  |  |
|  | 31.25 | 0.255 ± 0.005 | 90.1 | 9.9 |  |  |
|  | 15.62 | 0.285 ± 0.002 | 100 | 0 |  |  |

**Table S7. Cytotoxicity evaluation of ALUP, BA, and LUP at various concentrations against PANC-1 cancer cell line**

| ID | Conc. (ug/ml) | Mean O.D | Viability % | Toxicity % | IC50 (µg/ml) | IC50 (µM) |
| --- | --- | --- | --- | --- | --- | --- |
| PANC-1 | --- | 0.328 ± 0.007 | 100 | 0 |  |  |
| ALUP | 1000 | 0.073 ± 0.008 | 22.2 | 77.7 | 668.5 | 1.43 |
|  | 500 | 0.18 ± 0.008 | 55.9 | 44.1 |  |  |
|  | 250 | 0.32 ± 0.001 | 98.2 | 1.82 |  |  |
|  | 125 | 0.33 ± 0.002 | 100 | 0 |  |  |
|  | 62.5 | 0.33 ± 0.003 | 100 | 0 |  |  |
|  | 31.25 | 0.33 ± 0.001 | 100 | 0 |  |  |
| BA | 5000 | 0.018 ± 0.001 | 4.90 | 95.1 | 291.4 | 0.638 |
|  | 2500 | 0.021 ± 0.001 | 5.90 | 94.1 |  |  |
|  | 1250 | 0.023 ± 0.001 | 6.26 | 93.7 |  |  |
|  | 625 | 0.077 ± 0.004 | 21.1 | 78.9 |  |  |
|  | 312.5 | 0.165 ± 0.007 | 44.9 | 55..04 |  |  |
|  | 156.25 | 0.301 ± 0.004 | 82.10 | 17.9 |  |  |
| LUP | 5000 | 0.019 ± 0.001 | 5.17 | 94.8 | 402.6 | 0.944 |
|  | 2500 | 0.023 ± 0.001 | 6.26 | 93.7 |  |  |
|  | 1250 | 0.023 ± 0.001 | 6.26 | 93.7 |  |  |
|  | 625 | 0.082 ± 0.005 | 22.34 | 77.6 |  |  |
|  | 312.5 | 0.183 ± 0.005 | 50.04 | 49.95 |  |  |
|  | 156.25 | 0.345 ± 0.005 | 94.0 | 6.0 |  |  |
| Doxorubicin | 1000 | 0.03 ± 0.002 | 9.1 | 90.9 | 89.31 |  |
|  | 500 | 0.042 ± 0.001 | 12.5 | 87.5 |  |  |
|  | 250 | 0.07 ± 0.003 | 20.9 | 79.1 |  |  |
|  | 125 | 0.109 ± 0.003 | 32.3 | 67.6 |  |  |
|  | 62.5 | 0.19 ± 0.005 | 58.1 | 41.9 |  |  |
|  | 31.25 | 0.282 ± 0.002 | 83.7 | 16.3 |  |  |
|  | 15.62 | 0.33 ± 0.002 | 98.1 | 1.88 |  |  |
